# Extracellular vesicles from serum samples of mycobacteria patients induced cell death of THP-1 monocyte and PBMC

**DOI:** 10.1186/s12890-022-01839-w

**Published:** 2022-02-09

**Authors:** Alireza Javadi, Masoud Shamaei, Payam Tabarsi, Masoumeh Nomani, Mohammad Varahram, Bahram Kazemi

**Affiliations:** 1 Virology Research Center, National Research Institute of Tuberculosis and Lung Diseases (NRITLD), Shahid Beheshti University of Medical Sciences, Tehran, Iran; 2 Clinical Tuberculosis and Epidemiology Research Center, National Research Institute of Tuberculosis and Lung Diseases (NRITLD), Shahid Beheshti University of Medical Sciences, Tehran, Iran; 3 Mycobacteriology Research Center, National Research Institute of Tuberculosis and Lung Diseases (NRITLD), Shahid Beheshti University of Medical Sciences, Tehran, Iran; 4 Cellular and Molecular Biology Research Center, Shahid Beheshti University of Medical Sciences, Tehran, Iran

**Keywords:** Extracellular vesicles (EVs), Mycobacteria, Cell death, THP-1 monocyte, PBMC

## Abstract

**Background:**

Extracellular vesicles (EVs) play a key role in cell communication and the pathogenesis of some diseases. EVs may accelerate cell death during the course of mycobacterial infection and are also considered as a new vaccine design, drug delivery, and biomarker candidates. The current study evaluates the effects of EVs from serum samples of mycobacteria-infected patients on THP-1 monocytes and PBMC cells.

**Method:**

EVs were purified from the serum, then cultured separately with THP-1 monocytes and PBMCs. The cell death was determined through annexin V-FITC and PI staining. GW4869, an EVs inhibitor, was used to determine if EVs released from serum could increase THP-1 monocytes cell death.

**Results:**

The cell death was significantly increased in the presence of 10 µg/ml and 5 µg/ml concentrations of the purified EVs (*p* < 0.05). Minimal cell death was determined in 2.5 µg/ml and 1.2 µg/ml (*p* < 0.05). Up to 85% of the cells were viable in the presence of the GW4869 inhibitor (*p* < 0.05).

**Conclusion:**

Direct infection of the cells with EVs released from mycobacteria-infected patients samples, the multiplicity of infection with the EVs, and virulent or avirulent mycobacteria may change the status of the cell death. The isolated EVs  from serum samples of patients with mycobacterial  infection accelerated cell death, which means that they might   not be considered as an optimal tool for developing drug delivery and vaccine against tuberculosis.

## Background

The world still faces tuberculosis (TB), which is  one of the top ten causes of mortality and the cause of  a single infectious particle, as two billion people suffer from it [1]. Totally, the WHO has reported 1.4 million deaths from TB (including 208 000 patients co-infected with HIV) and 7.1 million people newly diagnosed cases of TB in 2019 [2, 3]. The frequencies of environmental *Mycobacterium*  in many geographical areas is affected by  higher prevalence of  *Mycobacterium* *Tuberculosis*. A significant proportion of non-tuberculous mycobacteria (NTM) are related to systemic or acquired immunodeficiency like infection with human immunodeficiency virus (HIV) [4].

Extracellular vesicles (EVs) are membrane-bound with a size of about 30–150 nm released by fusion of multivesicular bodies (MVB) with the cell plasma membrane [5]. They play a role in biology communication between cells and diverse pathogenesis of diseases as release from B cells and antigen presenting cells; including macrophages, dendritic cells (DCs), and natural killer cells. EVs are enriched with  proteins of  the tetraspanins family including CD63 and CD81 [6, 7] and molecules invovled in antigen-presentation to activated T cells (CD80, CD86, and MHC-II) [8].

EVs are carriers of tumor antigens and stimulate an antitumor response in mice [9, 10], which is a possible role for designing antitumor vaccines in human trials [11–13].  Additionally, the differences between EVs content in healthy and patient samples make it promising candidates for biomarker. EVs concentration in serum of infected mice with BCG or *M. tuberculosis* has been significantly higher in comparison with controls, suggesting EVs as a biomarker [14, 15]. More recently, studies have shown that tumor cells relaese EVs to accelerate tumor epithelial-mesenchymal transition, angiogenesis, and immune escape. The interaction between programmed cell death ligand-1 (PDL-1) and its receptor, programmed cell death 1 (PD-1), inhibits T cell responses, making EVs effective for immunotherapy of different cancers. Studies also have shown that PDL-1-releasing EVs inhibit anti-tumour immune responses [16]. Exosomal PDL-1 may target T cells and induce cell death [17]. In patients with melanoma, exosomal PDL-1 is also could be a marker of the clinical response to PD-1 blockage [16].

Once  the *Mtb * invades the host cells, it would be lysed in macrophages, and its contents are degraded into short-peptide segments. By releasing EVs, the complex formed through short-peptides binding to MHC-I and MHC-II molecules on the surface of EVs and transported to the surface of macrophages in order to activate macrophages or CD8 + and CD4 + T lymphocytes. Thus, a specific immune response against *M. tuberculosis* will be initiated in consequence [18, 19]. Studies indicate that EVs released by infected cells could mediate the inhibition of immune responses by accelerating cell death of immune cells. By causing the cell death  of the primary host cell, mycobacteria could control the infection [20]. The modulatory of EVs released from Mtb-infected macrophages has been reviewed [19] and induced cell death by EVs in recipient cells has been evaluated [21, 22].

The use of EVs as cell to cell communication for vaccines development, drug delivery, and biomarkers should be more analysed for a better understanding of how the EVs from infected cells alter immune responses, particularly the cell death. Hence, in vitro, different studies on immune response and cell death caused by EVs derived from macrophages infected with BCG and *Mycobacterium avium* have been reported, but no studies have been evaluated EVs from the patients with *Mtb* comparing with  non-tuberculous mycobacteria that may induce cell death. The current study presents cell death of THP-1 monocyte cells and peripheral blood mononuclear cells (PBMCs) by releasing EVs from the patients with mycobacterial infection.

## Methods

### Patient characterisations

In this study, we selected six diagnosed patients with non-tuberculous mycobacteria (NTM) species (including *M. abscessus, M. kansasi,* *M.* *chelonae, M. simiae, and M. avium)* and *Mycobacterium tuberculosis* aged from 32 to 83 years who were admitted at the Masih Daneshvari Hospital, a referral centre for tuberculosis and lung disease in Iran_Tehran, during 2018–2020. A healthy control group was also recruited for analysis of cell death by EVs. The inclusion criteria were positive mycobacterial culture, considering clinical and radiological findings corresponding mycobacterial infection (Table 1). In the current study, healthy individuals aged from 30 to 82 were also analysed for EVs cell death. All of the patients and controls were tested for HIV infection, healthy individuals had negative history of TB, or any chronic disease.

**Table 1:** Clinical characteristics of patients with mycobacterial infection

Type of bacteria	Age of patient	Gender	Cough, sputum and fever	HIV, malignancy or other infectious diseases	Diabetes mellitus	Hemoptysis	Smoking	Method of diagnosis
*Mycobacterium avium*	64	Male	Positive	Negative	Negative	*No*	*No*	Culture, AFB smear, RFLP PCR
*Mycobacterium abscessus*	53	Female	Positive	Negative	Negative	*No*	*No*	Culture, AFB smear, RFLP PCR
*Mycobacterium kansasi*	51	Male	Positive	Negative	Negative	*No*	*No*	Culture, AFB smear, RFLP PCR
*Mycobacterium chelonae*	32	Female	Positive	Negative	Negative	*No*	*Yes*	Culture, AFB smear, RFLP PCR
*Mycobacterium simiae*	83	Male	Positive	Negative	Negative	*No*	*No*	Culture, AFB smear, RFLP PCR
*Mycobacterium tuberculosis*	51	Male	Positive	Negative	Negative	*No*	*Yes*	Culture, AFB smear, RFLP PCR

Italics indicate Non-tuberculous mycobacterium (NTM) species and *Mycobacterium tuberculosis (Mtb)*

### Extracellular vesicles (EVs) isolation and characterization

Extracellular vesicles were isolated from serum samples of the patients infected with *Mycobacterium tuberculosis*, anontuberculous mycobacteria (*NTM*) patients and healthy individuals (controls). The serum EVs from the patients and controls were extracted using exoEasy Maxi Kit (Qiagen, Valencia, CA, USA, Cat. no. 76064) according to the manufacturer’s instructions [23]. Briefly, pre-filtering the serum samples to exclude cell contamination was performed using a 0.22 μm filter prior to extract EVs (Merck-Millipore, Billerica, MA, USA). Then, 1 volume (1000 µl) of buffer XBP was added to 1 volume (1000 µl) of the sample, mixed, and left to warm up at room temperature. The mixture was then transferred to the exoEasy spin column and centrifuged at 500×*g* for 1 min followed by discarding flow-through and placing the column into the collection tube. Next, 10 ml buffer XWP was added to the column and centrifuged at 3000×*g*. After discarding flow-through, 800 µl of buffer XE was added and incubated for 1 min, centrifuged at 500×*g* for 5 min and the flow-through was collected as EVs. EVs were kept at − 70 °C for the further analysis of size and morphology by flow cytometry assays (FACS Calibur, BD, USA). For bead-based flow cytometry method using magnetic beads coated with antibodies against tetraspanin CD81, 0.5 mg of purified EVs was coupled with 5 μm of aldehyde/sulfate latex beads (Thermo Fisher) and shacked overnight at 4 °C. The binding sites were incubated with 100 mM glycine (Sigma) for 30 minute, and then stained with CD9, CD63 and CD81 antibodies as detectable by the cytometer [24, 25]. The results were prepared by flow cytometer (FACS Calibur, BD, USA). The quantification and size analysis of the vesicles was done by electron microscopy (TEM Carl  Zeiss- EM10C-100 kV, Germany), dynamic light scattering (DLS).

### Peripheral blood mononuclear cells (PBMC) and THP-1 cells culture with extracellular vesicles of mycobacteria and healthy control

The human acute monocytic leukemia cell line THP-1 (ATCC, catalogue number: ATCC® TIB-202™, USA) and PBMCs from a healthy control were separately grown in RPMI 1640 (Gibco™; Carlsbad, CA, USA) supplemented with 10% heat-inactivated fetal bovine serum (FBS) (Gibco™), 25 mM HEPES (Gibco™), 100 units/ml penicillin (Sigma, Munich, Germany) and 100 μg/ml streptomycin (Sigma, USA), incubated for 24 h at 37 °C under 5% CO_2_. PBMC were isolated using density gradient centrifugation using Ficoll-Paque (Invitrogen Corp., Carlsbad, CA). Once cell culture has proliferated and confirmed by fluorescent microscopy, a separate experiment was initiated for those of EVs -derived from each mycobacterial species and healthy controls. In order to classify dose-dependent of EVs cell death with THP-1 monocyte cell and PBMC, based on our bicinchoninic acid assay findings (Pierce BCA Protein Assay (µg/ml)—Thermo Fisher Scientific Inc., no. 23225, USA), each well was separately coated with 200,000 cells/ml inoculated with 10 µg/ml, 5 µg/ml, 2.5 µg/ml, 1.2 µg/ml the EVs, and EVs-depleted FBS (Gibco™ A25904DG, USA) to the final volume of 200 µl of the wells. Each of those EVs concentrations were over three times performed. For each experiment, lipopolysaccharides (LPS, 100 ng/ml) and unstained controls were included. Then, the cells were incubated at 37 °C under 5% CO_2_ for 24 h. Following the incubation, the plates were centrifuged at 40×*g* for 5 min. The pellet was used for cell death assay using annexin V-FITC (Invitrogen, 0.25 µg/mL) and Propidium iodide (PI) (Invitrogen- Thermo Fisher Scientific Inc., Lot: 1989095, USA) by a flow cytometer (FACS Calibur, USA). Pellets were precipitated with the annexin V for 15 min in the dark at room temperature. Then, 10,000 events were evaluated at channels FL1, FL2 and cell death was determined.

### Extracellular vesicles inhibition assay via GW4869

One of the hypotheses of the current study is that accelerating cell death of THP-1 cells by EVs released from the serum of tuberculosis and non-tuberculous mycobacteria (NTM) patients may be decreased or inhibited via GW4869 and this may increase our understanding of EVs is a key reason of inducing cell death. Accordingly, peripheral blood mononuclear cells (PBMCs) from a patient with *mycobacterium intracellular* were isolated by density gradient centrifugation using Ficoll-Paque (Invitrogen Corp., Carlsbad, CA). After counting the cell, 120 µl of RPMI 1640 (Gibco; Carlsbad, CA, USA) supplemented with 10% heat-inactivated fetal bovine serum (FBS) (Gibco), 25 mM HEPES (Gibco™), 100 units/ml penicillin (Sigma, Munich, Germany) and 100 μg/ml streptomycin (Sigma, USA) was transferred to each well. In order to observe dose-dependent of cell death in the presence of EVs inhibitor (GW4869, D1692-Sigma-Aldrich, USA) [26], different concentrations of PBMC from the M. intracellular patient consisted of 10 µg/ml, 5 µg/ml, 2.5 µg/ml, 1.2 µg/ml (80 μl, 40 μl, 20 μl and 10 μl respectively) were added to the medium (two wells were repeated for each concentration). Then, GW4869 with a final concentration of 20 µM was added (1.6 µg/ml, 0.8 µg/ml, 0.4 µg/ml, 0.2 µg/ml, respectively). Plates containing the cells were incubated for 24 h in an incubator at 37 °C with 5% CO_2_. Then, the plate was centrifuged at 185×*g* for 5 min. The supernatant was kept at − 70 °C and the pellet was mixed with the annexin V-FITC (Invitrogen**,** 0.25 µg/mL) placed in a dark place for 15 min at room temperature. Next, 10,000 events were screened at channels FL1, FL2 and cell death was analysed. For each experiment, unstained controls were included.

### Statistical analysis

Data presented as multiple t-tests and graphs were generated using GraphPad Prism 6 software. *P*-value < 0. 05 was considered statistically significant.

## Results

### Characterization of extracellular vesicles (EVs)

**Fig. 1:**
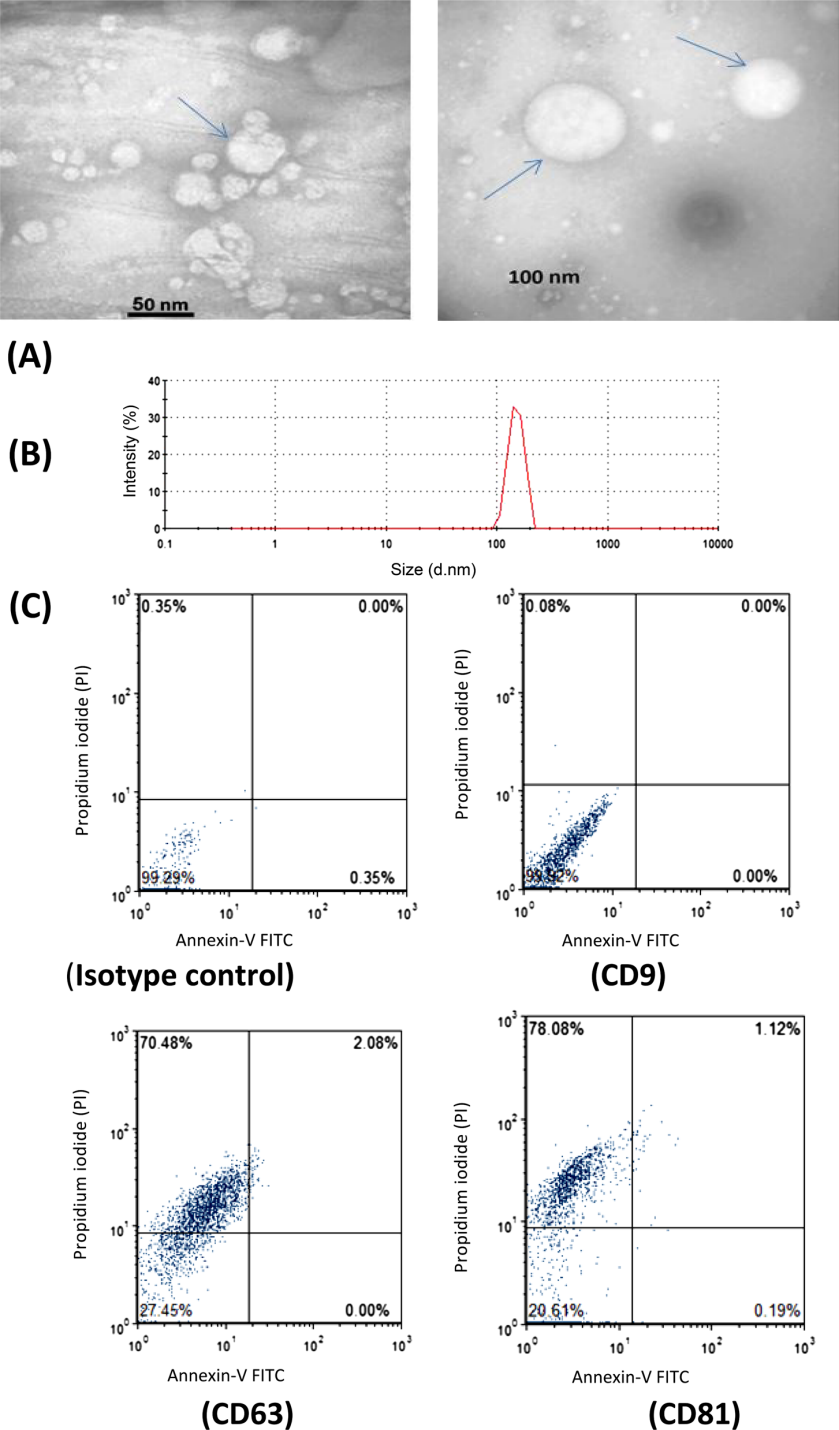
**A**–**C**: EVs characterization by transmission electron microscopy (TEM), Dynamic light scattering (DLS), and flow cytometer analysis. **A** TEM showing serum EVs as spherical particles with an average size of 50–100 nm. **B** DLS shows the size distribution of the EVs as 147.6 ± 3.4 nm. **C** Detection of EVs enriched proteins, CD9 (negative, 0.08%), CD63 (positive, 70.48%) and CD81 (positive, 78.08%) by flow cytometer

Extracellular vesicles (EVs) were isolated from human serum of the patients infected with *Mycobacterium tuberculosis (Mtb)*, nontuberculous mycobacteria (NTM)) species, and healthy individuals as controls using exoEasy Maxi Kit according to manufacturer’s structure and confirmed by transmission electron microscopy (TEM). The results were characterized with size distribution by dynamic light scattering (DLS). Serum EVs are found as spherical particles with an average of 50–150 nm in size. The expression levels of enriched proteins including CD9, CD63 and CD81 were determined by flow cytometer (Fig. 1A–C).

### Cell culture of peripheral blood mononuclear cells (PBMC) and THP-1 monocyte with extracellular vesicles isolated from mycobacteria patients

THP-1 monocyte cells and PBMCs were grown after 24 h incubation with RPMI 1640 (Gibco™; Carlsbad, CA, USA) supplemented with 10% heat-inactivated fetal bovine serum (FBS) (Gibco™), 25 mM HEPES (Gibco™), 100 units/ml penicillin (Sigma, Munich, Germany) and 100 μg/ml streptomycin (Sigma, USA) at 37 °C under 5% CO_2_. Different concentrations of EVs (10 µg/ml, 5 µg/ml, 2.5 µg/ml, 1.2 µg/ml) derived from serum sample patients of the 6 mycobacteria were separately cultured with THP-1 cells and PBMC inoculated with EVs-depleted FBS (Gibco™ A25904DG, USA), LPS (100 ng/ml).

### Cell death of THP-1 monocyte cells and PBMCs by EVs derived from mycobacteria

**Fig. 2:**
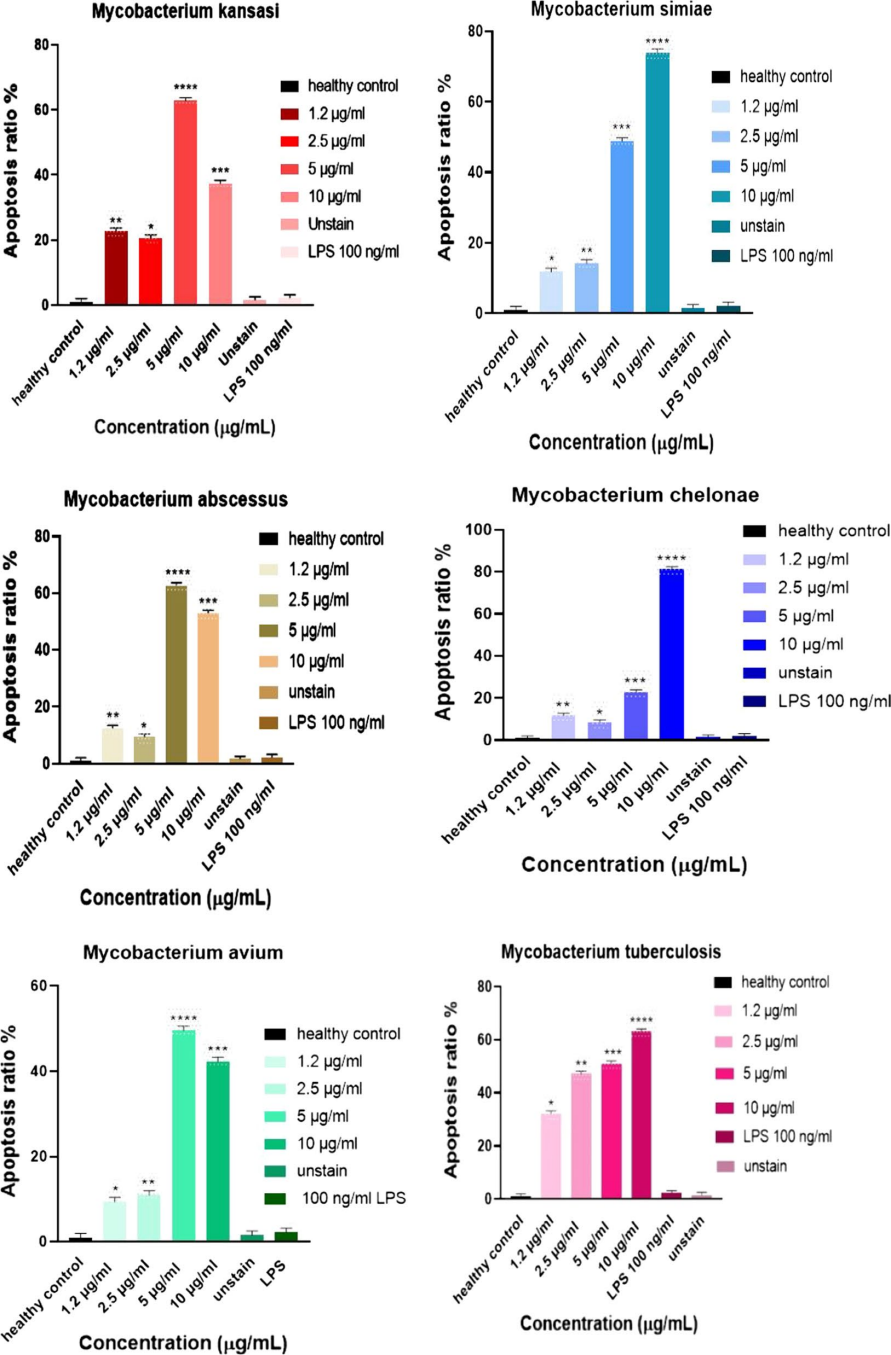
EVs induced cell death of THP-1 monocyte cells. Most cell death of THP-1 monocytes was found by increasing concentrations of EVs derived from mycobacteria. Minimum concentrations of the EVs induce  lowest cell death of THP-1 monocytes. Cell death was increased in the highest EVs concentrations and was decreased in the minimum of the EVs concentrations. **** ≤ 0.000001, *** ≤ 0.00001, ** ≤ 0.0001, * ≤ 0.001

**Fig. 3:**
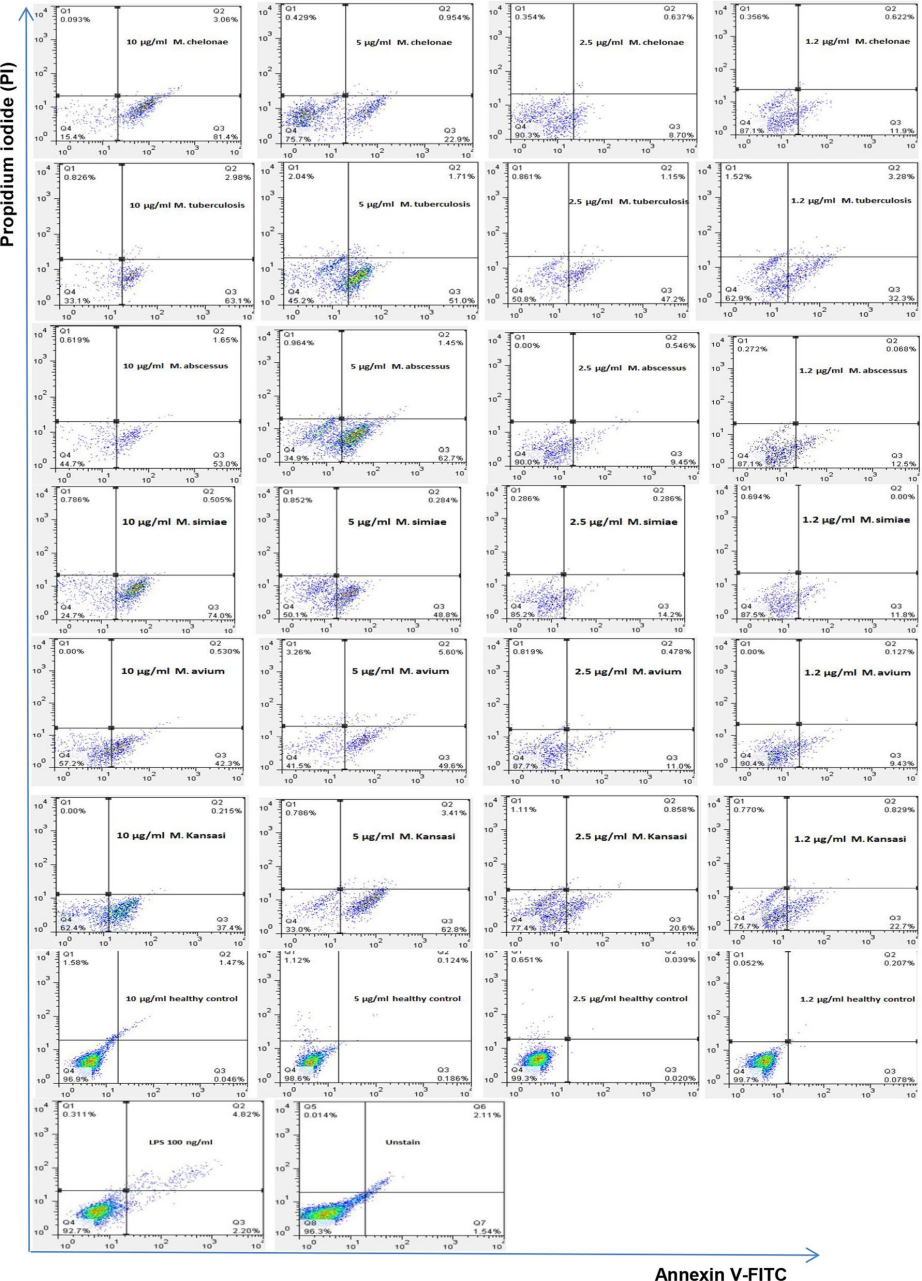
Flow cytometer data of mycobacteria EVs induced THP-1 monocytes cell death. THP-1 monocyte cells treated with mycobacteria EVs (10 µg/ml, 5 µg/ml, 2.5 µg/ml and 1.2 µg/ml), unstain cell, LPS, and healthy subjects. In most mycobacteria species, THP-1 monocytes cell death was increased by enhancing mycobacteria EVs multiplicities. *P* -value < 0.05 was considered

**Fig. 4:**
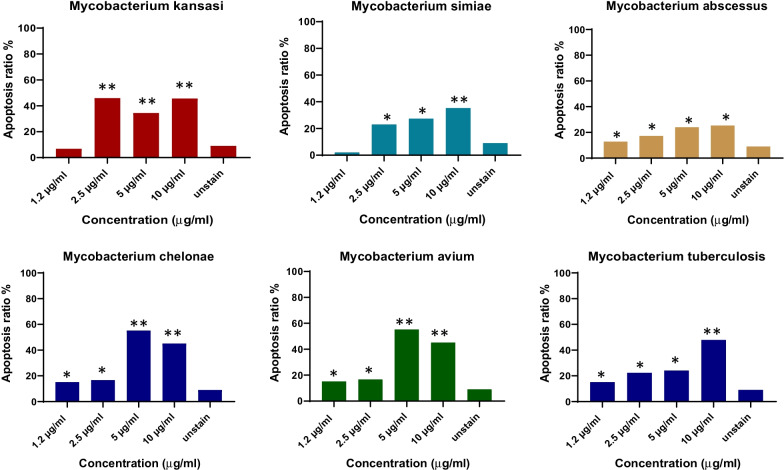
EVs from mycobacteria species induced cell death of healthy PBMCs. The most PBMCs cell death was found by increasing concentrations of the EVs. The cell death was increased in highest EVs concentrations 10, 5 and 2.5 µg/ml of the EVs concentrations. **P value < 0.05 was considered

**Fig. 5:**
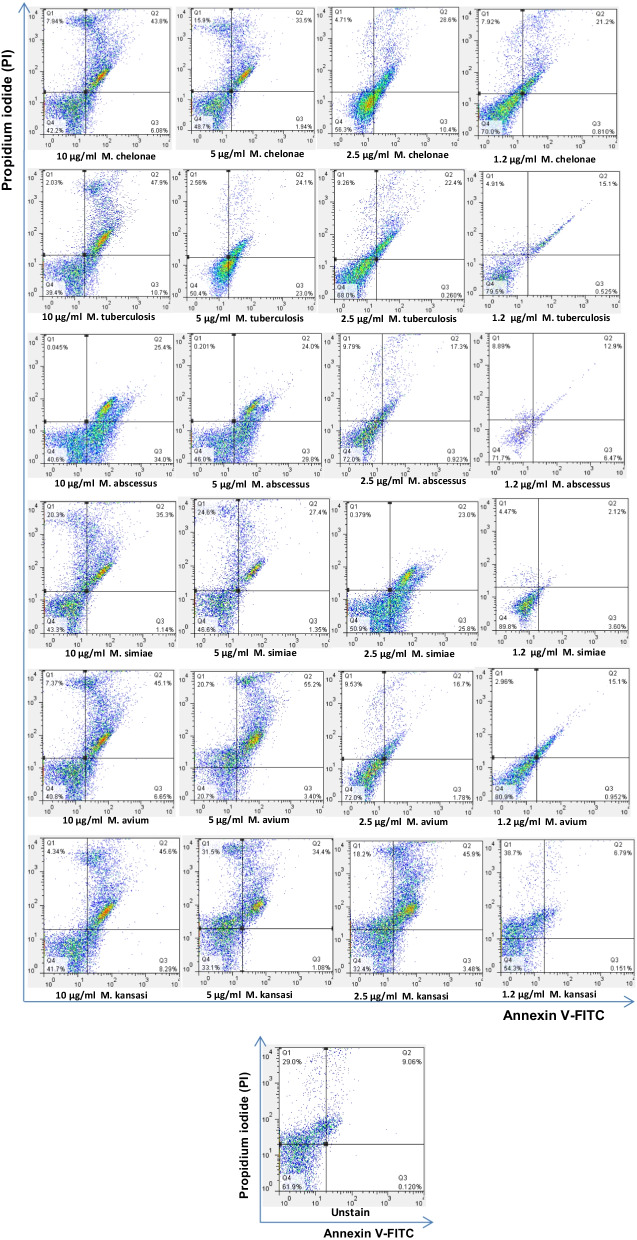
Flow cytometer analysis of the cell death by mycobacteria EVs on PBMCs from healthy control. PBMCs treated with different concentrations of EVs derived from mycobacteria. In most mycobacteria species, the cell death of PBMCs accelerated by increasing mycobacteria EVs multiplicities. *P*-value < 0.05 was considered

The cell death of THP-1 monocytes and PBMC cells was induced by EVs from serum samples of *Mycobacterium tuberculosis (Mtb) and Non-tuberculous mycobacteria (NTM)* patients. The cell death was performed in different concentrations of serum- EVs and assessed by annexin V-FITC and Propidium Iodide (PI) staining. Most cell death of THP-1 monocytes and PBMC cells were found in 10 µg/ml and 5 µg/ml concentrations of the EVs (*p* < 0.05). However, minimum cell death was determined in 2.5 µg/ml and 1.2 µg/ml (*p* < 0.05) (Figs. 2, 3, 4, 5). Also, cell death by EVs derived from healthy controls was analysed. The cells were viable in different concentrations EVs derived from serum samples of healthy control.

### EVs inhibition via GW4869

**Fig. 6:**
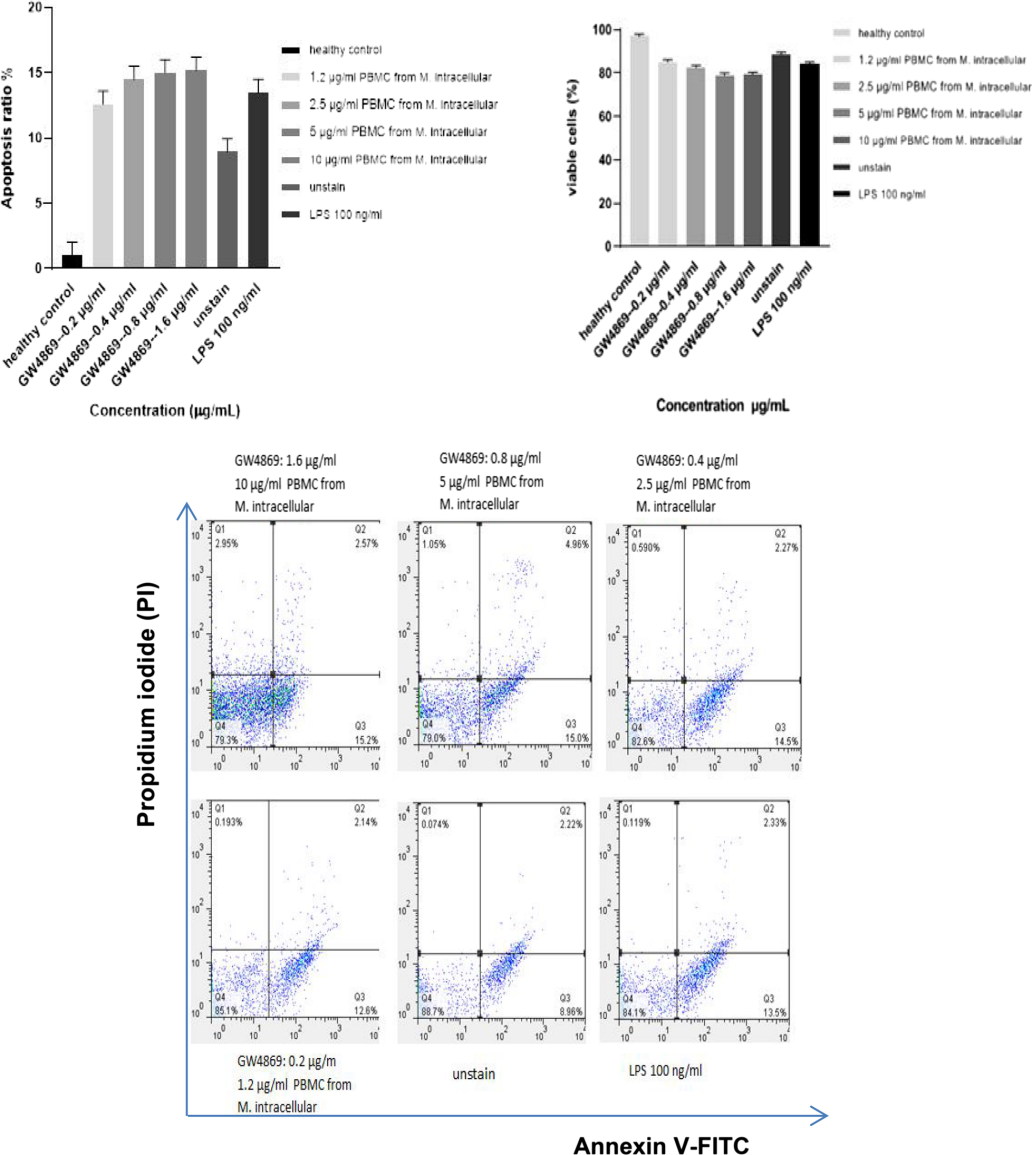
Flow cytometer data of EVs inhibition via GW4869. Different concentrations of PBMC from a patient with M. intracellular were mostly viable in the presence of GW4869 as the EVs inhibitor. The cell death of the PBMC was reduced in the presence of GW4869. *P*-value < 0.05 was considered

The cell death of different concentrations of PBMCs derived from the serum of patient infected with *mycobacterium intracellular* was also analysed in the presence of EVs inhibitor GW4869 in order to confirm whether cell death is induced by EVs directly released from mycobacteria or not. Up to 85% of the cells were viably kept in the presence of the inhibitor. Approximately, 15% of the cell death was observed by annexin-V/PI staining (Fig. 6). *P*-value < 0.05 was found compared to the healthy subject.

## Discussion

Extracellular vesicles (EVs) act as carriers of pathogen origin serving as antigens to induce host defense and immunity, or regulators of host defense, mediators of immune evasion particularly cell death [20]. This study evaluates the role of EVs released from serum samples of patients with different mycobacterial infection on the cell death of the THP-1 monocyte cells and peripheral blood mononuclear cells (PBMCs). The main finding of the current study was that EVs isolated from the serum samples of infected patients with *Mtb* and non-tuberculous mycobacteria (NTM) may associate with cell death of THP-1 monocyte cells and PBMCs. Therefore, since different purposes of EVs like in vaccine designing and diagnostic biomarkers have been assessed, cell death induced by EVs might change the way of developing a new vaccine and novel biomarkers [14]. In 2015, Wang et al. reported an association between EVs released from *M. avium*-infected macrophages with cell death [6]. They found that infected macrophages with EVs from *M. avium* are able to induce immune responses comparable with infection of the cells with *M. avium* only that not able to increase cell death. They also qualified the cytokine assay, and observed that only infection of the cell with *M. avium* induced high levels of TGF-β1 expression resulting to accelerate cell death. They concluded EVs trigger inflammatory responses in macrophages owing to the presence of bacterial antigens but have no effect on macrophage viability; suggesting that EVs may consider as a good vehicle for vaccine delivery [6]. However, one main drawback of their study could be the cells of the body have undergone many different factors in vivo including genetic differences and protein expression dysfunctions that might basically change cell death and cell viability, whereas the cells are controlled in vitro conditions. In 2013, Cheng and colleagues demonstrated  that EVs carrying mycobacterial antigens protect mice from *M. tuberculosis* infection. They revealed that EVs from macrophages treated with *M. tuberculosis* culture filtrate protein-treated (CFP-treated) were found to induce antigen-specific IFN-γ and IL-2-expressing CD4 + and CD8 + T cells [27]. The protection was equal or even superior to BCG. They concluded that EVs might serve as a novel cell-free vaccine against *M. tuberculosis* infection [27]. However, cell death could be assessed as the main factor of immune and cellular responses during a vaccine preparation. In 2010, Giri et al. described proteomic analysis of highly antigenic proteins on EVs from *M. tuberculosis*-infected and culture filtrate protein-treated macrophages [28]. They found about 29 types of *M. tuberculosis* proteins in EVs released from CFP-treated J774 cells and the most types were found in EVs only isolated from *M. tuberculosis*-infected cells. This suggests that EVs comprising *M. tuberculosis* antigens may be evaluated as a new approach to preparing a novel tuberculosis vaccine [28]. But, the J774 cells treated with CFP should be analyzed for cell death as inhibition of immune responses mainly increased by cell death of immune cells and EVs directly derived from mycobacteria and its interactions with the cells may show different outcomes for a novel vaccine. Also, in 2008, a work by Giri et al., reported EVs released from *M. Bovis* BCG infected macrophages were able to activate antigen-specific CD4 + and CD8 + T cells in both vitro and in vivo conditions [29]. They revealed that EVs from infected macrophages decrease deficiencies in antigen presentation related to mycobacterial infections and so this considers that EVs could be a great promising of *M. tuberculosis* vaccine development [29]. However, we thus believe that the work requires analyzing cell death to understand how EVs from *M. Bovis* BCG accelerates or decreases cell death as any change in cell death may result in modulation of immune responses by downregulating MHC II molecules. Also, cell death in vivo and in vitro must be analyzed as the body’s cells might show different cell death statuses by crosstalk between directly or indirectly derived EVs from mycobacteria and the cells.

In the current study, cell death of THP-1 monocytes and PBMCs cell was mostly found in the maximum concentrations (10 µg/ml and 5 µg/ml) of EVs released from mycobacteria species. But, minimum cell death was detected by the lowest EVs concentrations (2.5 µg/ml and 1.2 µg/ml). The most cell death of THP-1 was found in 10 µg/ml EVs from *M. chelonae*, however the lowest cell death were found in 2.5 µg/ml of the EVs released from *M. chelonae*. For PBMCs, the maximum cell death was seen in 10 µg/ml EVs from Mtb and NTM. A drawback of our study is that increasing cell death could be not only due to mycobacterial infections, because chronic diseases can also accelerate cell death, and EVs released from serum samples of the patients might show different outcomes and cellular responses on THP-1 monocytes and PBMCs in vitro in comparison with the cells in vivo. In this study, the function of EVs which induces cell death on PBMCs has been evaluated using GW4869 as an EVs releasing inhibition. The cell death was significantly reduced by different concentrations of PBMCs derived from *mycobacterium intracellular* in the presence of GW4869 EVs inhibition. Viable cells were determined in more than 85% of the cells in the presence of GW4869. Another limitation of the current study is that activation of caspase-3 and use of Z-VAD-FMK as a pan-caspase inhibitor to confirm if cell death is reduced, was unevaluated.

Studies have suggested that pathogenic mycobacteria species inhibiting cell death as a virulence mechanism are directly dependent on the multiplicity of infection and relative virulence of the strains [30]. Keane et al. reported that at low multiplicities of infection virulent *M. tuberculosis* induced less macrophage cell death than avirulent *M. tuberculosis* or other mycobacteria [31]. Therefore, it has been proposed that virulent *M. tuberculosis* inhibits cell death, while avirulent mycobacteria induce cell death. Some studies have suggested a variety of mycobacterial species [30] increase macrophages cell death in vitro. More recently, the interest has been based on the potential role of macrophages' cell death in host defense against mycobacterial infection [32]. Both monocytes and alveolar macrophages infected with *M. tuberculosis* have increased cell death in vitro, and a higher rate of cell death has been observed with alveolar macrophages isolated from pulmonary tuberculosis patients. Also, infection with low numbers of viable bacilli inhibits cell death in monocytes [33]. It has been reported that compared with relatively avirulent strains, infection of macrophages with virulent *M. tuberculosis* causes less cell death [31, 34]. Hence, based on these studies, an increased rate of cell death might be directly infected THP-1 monocytes and PBMC cells with EVs released from mycobacteria species resulting in TGF-β1 dysfunction and inducing TGF-β1 expression. A multiplicity of infection could be another consideration as lower multiplicities of mycobacterial infection-induced less cell death than higher multiplicities of  the mycobacterial infection that resultes necrosis-like cell death by a caspase-independent model. The current study found that 10 µg/ml and 5 µg/ml, both maximum multiplicities of EVs from mycobacteria increase the rate of THP-1 monocytes and PBMCs cell death compared with lower multiplicity including 2.5 µg/ml and 1.2 µg/ml. Another considerable cause of cell death could be either the model of virulent mycobacteria that decreases cell death or avirulent mycobacteria which induce cell death. Accordingly, this may be due to the fact that EVs from *M. chelonae* were shown to cause the lowest level of monocytes cell death, while different concentrations of EVs from *M. tuberculosis* caused the most monocytes cell death.

## Conclusion

The cell death can undergo different mechanisms including the  cells infected directly with extracellular vesicles (EVs) from mycobacteria species, the multiplicity of infection with EVs directly derived from those of virulent or avirulent mycobacteria. As the first observation, we believe that EVs from serum samples of mycobacterial infected patients have been associated with accelerated cell death and this may result in new advances in vaccine or drug developments against *Mtb*. To demonstrate if cell death is decreased, further studies need to be conducted involving the interaction between the cells and directly EVs purified from more individulas with mycobacterial infection, as well as  the use of pan-caspase inhibitors like Z-VAD-FMK to inhibit activation of caspases.

## Data Availability

The datasets used and/or analyzed during the current study are available from the corresponding author upon reasonable request.
